# Identification of PSD-95 in the Postsynaptic Density Using MiniSOG and EM Tomography

**DOI:** 10.3389/fnana.2018.00107

**Published:** 2018-12-07

**Authors:** Xiaobing Chen, Christine Winters, Virginia Crocker, Michael Lazarou, Alioscka A. Sousa, Richard D. Leapman, Thomas S. Reese

**Affiliations:** ^1^Laboratory of Neurobiology, National Institute of Neurological Diseases and Stroke, National Institutes of Health, Bethesda, MD, United States; ^2^EM Facility, National Institute of Neurological Diseases and Stroke, National Institutes of Health, Bethesda, MD, United States; ^3^Surgical Neurology Branch, National Institute of Neurological Diseases and Stroke, National Institutes of Health, Bethesda, MD, United States; ^4^Laboratory of Cellular Imaging and Macromolecular Biophysics, National Institute of Biomedical Imaging and Bioengineering, National Institutes of Health, Bethesda, MD, United States

**Keywords:** PSD-95, miniSOG, EM tomography, photoconversion, diffusion, labeling

## Abstract

Combining tomography with electron microscopy (EM) produces images at definition sufficient to visualize individual protein molecules or molecular complexes in intact neurons. When freeze-substituted hippocampal cultures in plastic sections are imaged by EM tomography, detailed structures emerging from 3D reconstructions reveal putative glutamate receptors and membrane-associated filaments containing scaffolding proteins such as postsynaptic density (PSD)-95 family proteins based on their size, shape, and known distributions. In limited instances, structures can be identified with enhanced immuno-Nanogold labeling after light fixation and subsequent freeze-substitution. Molecular identification of structure can be corroborated in their absence after acute protein knockdown or gene knockout. However, additional labeling methods linking EM level structure to molecules in tomograms are needed. A recent development for labeling structures for TEM employs expression of endogenous proteins carrying a green fluorescent tag, miniSOG, to photoconvert diaminobenzidine (DAB) into osmiophilic polymers. This approach requires initial mild chemical fixation but many of structural features in neurons can still be discerned in EM tomograms. The photoreaction product, which appears as electron-dense, fine precipitates decorating protein structures in neurons, may diffuse to fill cytoplasm of spines, thus obscuring specific localization of proteins tagged with miniSOG. Here we develop an approach to minimize molecular diffusion of the DAB photoreaction product in neurons, which allows miniSOG tagged molecule/complexes to be identified in tomograms. The examples reveal electron-dense clusters of reaction product labeling membrane-associated vertical filaments, corresponding to the site of miniSOG fused at the *C*-terminal end of PSD-95-miniSOG, allowing identification of PSD-95 vertical filaments at the PSD. This approach, which results in considerable improvement in the precision of labeling PSD-95 in tomograms without complications due to the presence of antibody complexes in immunogold labeling, may be applicable for identifying other synaptic proteins in intact neurons.

## Introduction

Molecular understanding of the function of synapses ultimately requires delineation of the three-dimensional (3D) organization of major synaptic molecules in their functional states. Electron tomography (ET) of synapses provides 3D reconstructions of the organization of molecular complexes in intact neurons at a spatial resolution of 2 – 4 nm (Lucic et al., 2005a; Chen et al., 2014). At the outset of the application of ET to neuroscience, the combination of tomography with conventional transmission electron microscopy (TEM) preparation produced remarkable images unveiling the organization of the presynaptic specialization in the fixed neuromuscular junction (Harlow et al., 2001). In those initial experiments, the use of heavy metal staining provided sufficient contrast in 3D reconstructions to reveal the shapes and locations of molecular complexes, but not their molecular identities since in addition to the presence of staining masking proteins, the tomographic resolution of 2 – 4 nm is insufficient to match with known high-resolution protein structures that would enable their identification. It remains a challenge to sort out definitively the molecular identities of structural complexes in tomograms of intact neurons.

Transmission electron microscopy tomography can be applied to cultured neurons or isolated synaptosomes from brain tissues prepared in the vitreous state by rapid freezing or hyperbaric freezing followed by cryosectioning or cutting into thin lamellae using a focused ion beam (FIB) (Lucic et al., 2005b, 2007; Rigort et al., 2012). With the recent improvements in direct electron detectors (Li et al., 2013) and phase plate technology (Fukuda et al., 2015), cryo-TEM has the potential to provide sufficient resolution and contrast to visualize macromolecular details in unstained samples without the need to average over multiple copies of the structure. Although frozen, vitrified cells are prepared close to their native state, thereby avoiding chemical fixation artifacts. Current cryo-electron tomography still falls short in terms of contrast and spatial resolution to identify many structural elements, but as discussed below, a pathway to tag molecules and to identify them by freeze-substitution and TEM tomography in neurons seems feasible.

The excitatory postsynaptic density (PSD) is a large macromolecular signaling assembly appearing as prominent electron-dense structure in the postsynaptic membrane apposing the presynaptic terminal at glutamatergic synapses (Kennedy, 2000; Sheng and Kim, 2011). Progress to date in TEM tomography of synapses, particularly at the PSD, has been facilitated by combining heavy metal staining with high pressure freezing and freeze-substitution to prepare tissues (Chen et al., 2008b,c, 2014; Imig et al., 2014; Korogod et al., 2015). Detailed data analysis from tomograms relating to the sizes, numbers (Chen et al., 2005; Sheng and Hoogenraad, 2007) and orientations of the PSD components (Chen et al., 2008b, 2011) has made it possible to deduce the molecular identities of structures within the PSD, including glutamate receptors such as NMDA, AMPA receptors, and PSD-95 MAGUKs—the family of major scaffolding proteins anchoring glutamate receptors at the PSD (Chen et al., 2005, 2008b, 2011, 2015). These identifications were tested using acute protein knock-down or gene knock-out of these synaptic components prior to high pressure freezing and freeze substitution (Chen et al., 2011, 2015; Imig et al., 2014). Identification of these structures relies on TEM tomography of intact PSDs, followed by detailed analysis of structural changes observed in tomograms in knockdown experiments (Chen et al., 2011, 2015). Identifying molecules or molecular complexes by this approach has offered unique insights, but it is indirect in the sense that loss of a key structural protein could lead to many changes in a complex structure like the PSD.

Seeking a more direct method to label structures identifiable in tomograms, we have experimented with immunolabeling using Nanogold (1.4 nm) attached to a secondary antibody applied to lightly fixed specimens, which were then freeze-substituted after controlled silver enhancement of the gold nanoparticles to render them a useful size and density for detection by TEM (Chen et al., 2008a,b, 2014). Even with the initial chemical fixation, many, but not all of the fine structural details in the PSD structure are retained and recognized. One can expect to label only a small fraction of any population of molecules with antibodies, and the presence of antibody complexes leaves the silver enhanced gold particles separated by at least 10–20 nm from their target sites (Zhang et al., 2015), complicating the unambiguous identification of target structures in tomograms. However, this antibody method has proven useful for identifying PSD-95 molecules at the PSD, where they appear to be uniformly oriented and clustered, making it more apparent which structures are labeled (Chen et al., 2008a, 2011).

A major breakthrough occurred in 2011 with the publication of a seminal paper entitled, *A genetically encoded tag for correlated light and electron microscopy of intact cells, tissues, and organisms* (Shu et al., 2011), which held promise as a tool for labeling molecules in TEM with a higher degree of specificity than conventional immunolabeling. Like immunolabeling, this new approach depends on chemical fixation to stabilize the tissue while activating the label, but some labeled structures can still be identified at the EM level. MiniSOG (mini singlet oxygen generator), as the new probe was named, employs a designer *Arabidopsis* peroxidase moiety to generate free singlet oxygen molecules and precipitate diaminobenzidine (DAB) polymer, which can then be intensely stained with OsO_4_.

Horseradish peroxidase (HRP), long used as a space tracer in tissues, was subsequently used to show the exact location of the blood-brain barrier in the vascular endothelium of the brain (Reese and Karnovsky, 1967; Brightman and Reese, 1969). Recently developed versions of cloneable HRPs such as APEX and APEX2, can provide more localized labeling of cellular structures in the proximity of APEX/APEX2 tagged proteins, though not yet at the level needed to identify molecules/complexes in cells (Martell et al., 2012; Lam et al., 2015). Unfortunately, it quickly became apparent that DAB photoreaction product generated by peroxidase undergoes considerable local diffusion before it precipitates (Courtoy et al., 1983; Brown and Farquhar, 1989). The reaction product, however, does not readily cross membranes, so miniSOG has proven useful to designate membrane compartments. Although photooxidation has been done at low temperature to dampen diffusion (Deerinck et al., 1994; Horstmann et al., 2013), no effective solution to minimizing diffusion in cytoplasm has been described. When PSD95-miniSOG was overexpressed in synapses, its photoreaction product (DAB polymer) was distributed throughout the entire cytoplasm of the dendritic spine, making it impossible to identify individual PSD-95 molecules at the PSD. We sought to develop means to suppress diffusion of DAB so that the technique could be used to identify individual structures in the tomograms of tissue expressing miniSOG. Here we describe our initial successes with this approach in the hope that it will pave the way for further development of the miniSOG technique to better label individual molecules at synapses and elsewhere.

## Materials and Methods

### Animals and Neuronal Culture

This work was approved by the National Institute of Neurological Disorders and Stroke (NINDS)/The National Institute on Deafness and Other Communication Disorders (NIDCD/The National Center for Complementary and Integrative Health (NCCIH) Animal Care and Use Committees (ACUC).

Rat hippocampal neurons prepared from E20 rat embryos were layered onto confluent glial feeder cultures that had been plated onto 25 mm round German glass coverslips (Mayer et al., 1989). The coverslips had been previously cleaned in nitric acid and coated with poly-L-lysine (Sigma) before plating.

Neuronal cell cultures were fed three times weekly with half changes of MEM (containing Earle’s salts, 6 g/l glucose, 3.7 g/l sodium bicarbonate) supplemented with 5% (v/v) heat-inactivated horse serum, 2% (v/v) fetal bovine serum, 2 mM Glutamax (all from Life Technologies), 136 μM Uridine 54 μM 2-deoxy-5-fluoro-uridine (Sigma), and N3 supplement [BSA, apotransferrin, putrescine, selenium, T3, insulin, progesterone, corticosterone (Sigma) (Ransom et al., 1977)]. Cells were maintained in a 36°C incubator with 10% CO_2_.

### Construct and Transfection

The PSD-95-miniSOG plasmid was built from PSD-95-EYFP (Chen et al., 2011) by switching the *EFYP* to *miniSOG*, which was obtained from the laboratory of Roger Tsien (UCSD). Dissociated rat hippocampal neurons (E20) were grown directly on a glia layer on 25 mm glass cover slips (Warner Instruments) for 21 days before being transfected with the PSD-95-miniSOG construct using Clontech CalPhos Mammalian Transfection Kit (Chen et al., 2011), typically 2–5 μg cDNA per coverslip was used for transfection, and followed by 18–20 h of incubation for the protein expression.

### Photoconversion

The miniSOG transfected cells were rinsed with modified Krebs Ringer, fixed with 2% glutaraldehyde (or 2% acrolein) in 0.1 M cacodylate buffer for 30 min, and then rinsed in 0.1 M sodium cacodylate buffer at pH 7.4 blocked for at least 30 min in 50 mM glycine, 10 mM potassium cyanide, and 5 mM 3-Amino-1,2,4-triazole (Shu et al., 2011). The round coverslip with fixed cells was transferred to a Warner chamber and sealed. All photoconversion procedures were carried out in the dark on a confocal microscope. Initial quick scan of the neurons using 488-nm excitation at low magnification was performed at low laser power to minimize photobleaching to locate the cells of interest. The temperature control was set through a custom-made metal device fitted on a Zeiss 20× water immersion objective with a water-circulating temperature controller (Lauda, RCS). The local temperature at the specimen was measured with a thermocouple thermometer. A specific cell or area of interest identified after quick scanning was illuminated with a maximum light intensity from an arc lamp passing a blue filter on a 510 Zeiss confocal microscope with a 20× objective during photoconversion. The photoconversion process was performed in the presence of solution of 1 mg/ml DAB in 0.1 M cacodylate or concentration of 20, 30, 40, and 60% (w/w) sucrose solution in 0.1 M cacodylate at room temperature (22°C) or at low temperature (∼4°C) in 40% (w/w) sucrose solution in 0.1 M cacodylate. The DAB solutions were bubbled with oxygen gas for about 20 min and then were passed through a 0.22 μm filter (Merck Millipore) before being added to cells. Cells were exchanged from cacodylate buffer to sucrose solution in cacodylate with DAB at room temperature and allowed 10–20 min to equilibrate, and then either photoconverted at room temperature or at a temperature of 4°C. When multiple cells were photoconverted in the same preparation, the equilibration time was longer, up to an hour. For low temperature photoconversion, the water bath temperature was set to 0.4°C and local temperature was measured with a thermocouple to be ∼4°C. Only 40% sucrose solution with DAB was used for low temperature photoconversion experiments. The time course was monitored with a timer in both transmission and fluorescent light channels. Before or after photoconversion, a diamond wedge mounted on the microscope stage was used to etch a ∼1.6 mm circle on the back of the cover slip to mark the area encircling the neurons that were to be or had been photoconverted. The cover slip was then rinsed in 0.1 M cacodylate and treated with 0.25 to 1% osmium tetroxide for 30 min on ice before further EM processing.

### EM Processing

The osmicated coverslip was further processed for EM: dehydration with one change at 7 min for each of 50, 70, and 90% EtOH, followed by three 7-min rinses with 100% EtOH; infiltration with Epon for 30 min of 1:1 Epon/EtOH; 30 min with 2:1 Epon/EtOH at room temperature; 10 min at 47°C with 100% Epon, and twice 1 h at 47°C with 100% Epon. Polymerization was in a 50°C oven overnight, and followed by transfer of the sample into a 60°C oven. The block with embedded coverslip was examined in a light microscope to mark the photoconverted area with the etched circle on the glass as a guide. The glass coverslip was released from the Epon block by brief immersion in liquid nitrogen and the marked area was cut out with a saw and mounted for thin sectioning. For conventional EM morphological analysis, the section was cut *en face* to a thickness of ∼70 nm and grid stained with UA and lead citrate. For TEM tomography, the sections were cut to a thickness of either ∼80 or ∼160 nm and mounted on Pioloform F coated 200 mesh hexagonal grids; some of them were grid stained for the experiments indicated and others were left unstained for TEM and EM tomography imaging.

### EM Tomography Data Acquisition, Tomogram Segmentation and Surface Rendering

Dual-axis TEM tomography series were acquired using FEI Tecnai TF30 TEM microscope operating at a beam voltage of 300 kV, equipped with a field emission gun and bottom-mounted 2 k × 2 k pixel CCD camera. The specimen tilt increment was 1.5 or 2°, and the typical tilt range was ± 70°, as limited by occlusion of the imaging field by the sample grid. Images from the dual-axis tilt series were processed, reconstructed and merged using IMOD package (Kremer et al., 1996). The 3D volume data were analyzed with EM3D (Harlow et al., 2001) (Stanford, CA, United States), segmented and surface-rendered in Amira (Thermo Fisher). Segmentation of tomograms, which are stacks of 2D slices of 3D reconstructions of synapses, were performed as described (Chen et al., 2008b,c, 2014). Briefly, visual inspection from consecutive virtual sections in tomograms allows identification of distinct classes of structures based on their sizes and shapes at the postsynaptic membrane. Each class of structure is manually traced by covering the electron-dense pixels regarded as belonging to the particular structure in all three orthogonal views in Amira, the consecutive contour lines are stacked up and surface-meshed to generate a surface rendering of the structural model. The size of particular structure can be measured from the surface-rendered model with built-in measurement tools such as the 3D ruler in Amira.

### EM Image Analysis

Line profiles of EM images were analyzed using the line profile tool in Fiji (ImageJ, NIH), which extracts the gray-level value of each individual pixel along any specific vectoral line (increased gray value from black to white). To normalize the line profile, the pixel gray value along the line was measured with Fiji, then each gray value was divided by the largest value within 20 nm from the starting point to ensure that all gray values were normalized to the background pixel value in the synaptic cleft, allowing gray level changes in different regions to be compared. Statistical analysis and data illustration were performed with GraphicPad Prism (La Jolla, CA, United States).

## Results

### Photoconversion of Overexpressed PSD-95-MiniSOG

**FIGURE 1:**
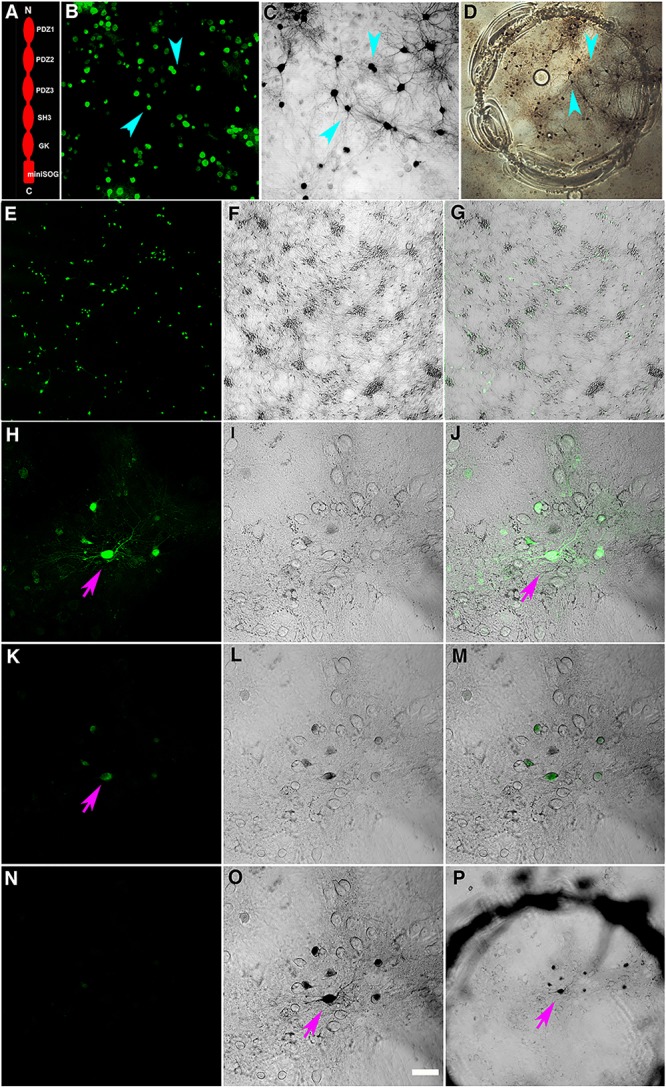
**(A)** Schematic drawing of PSD-95-miniSOG construct, made from PSD-95-EYFP (Chen et al., 2011) by replacing the extreme C-terminal fusion of *EYFP* with *miniSOG*. **(B)** Dissociated rat hippocampal neurons grown on a glass coverslip transiently transfected with and expressing green fluorescent PSD-95-miniSOG after glutaraldehyde fixation; note some non-transfected cells showed lower level of background fluorescence due to fixation. **(C)** Phase contrast transmission light micrograph showing darkened cells and processes following photoconversion in cacodylate buffer at 22°C. Cyan arrowheads point to specific miniSOG positive neurons in **(B,C)**. **(D)** Low magnification light micrograph showing an area on a glass coverslip etched with a diamond point containing photoconverted neurons. Cyan arrowheads point to the same neurons in **(B,C)**. **(E–G)** Low magnification light micrographs show PSD-95-miniSOG expressing neurons. **(E)** Fluorescent image of neurons expressing PSD-95-miniSOG. **(F)** Transmission phase contrast light micrograph showing neurons on a coverslip. **(G)** Overlay of **(E,F)** indicating percentage of neurons expressing miniSOG following transfection. **(H)** PSD-95-miniSOG positive neuron before photoconversion manifesting green fluorescence (purple arrow). **(K)** The same miniSOG expressing neuron in **(H)** loses 87% of original averaged intensity after 2 min photobleaching. **(I,L)** Transmission phase contrast light micrograph of the same region in **(H,K)**. **(J,M)** Overlay of **(H,I)** and **(K,L)** resulting **(J)** and **(M)**, respectively. **(N–P)** Neuron expressing PSD-95-miniSOG illuminated with blue light in **(H)** showing complete bleaching of fluorescent signal **(N)** and creating photoreaction products darkening neuronal cell bodies and processes in 7 min **(O)**. Scale bar, 50 μm **(H–O)**. **(P)** Low magnification phase contrast light micrograph of the area in **(H–O)** containing the photoconverted neurons and processes encircled by the mark indicating illuminated area on the glass coverslip.

Three-week (21 DIV) hippocampal neuron cultures grown on a glial feeding layer on glass coverslips were transfected with PSD-95-miniSOG construct (Figure 1A) and transfected neurons were incubated overnight and then fixed with glutaraldehyde or acrolein prior to photoconversion. Under 488-nm laser excitation, many glutaraldehyde-fixed cells exhibited low level green fluorescence, but the green fluorescence signal from the overexpressed PSD-95-miniSOG in the transfected cells (Figures 1E–G) was much stronger and readily distinguished from the background originating from non-transfected cells (Figures 1B,E,H). To start the photoconversion process, a neuron and its processes of interest were first identified (Figure 1H), and then the cell media was exchanged with a buffer containing a saturated solution of DAB and equilibrated before being illuminated at full power with light from a mercury arc lamp passing through a blue filter.

**FIGURE 2:**
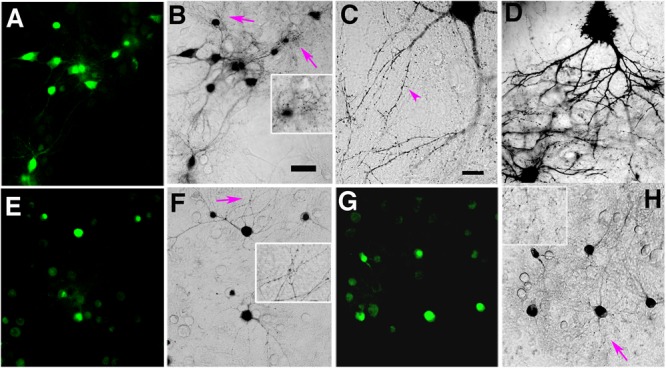
**(A–C)** Blue light illumination of miniSOG-expressing neurons in DAB photoreaction medium generates progressively more photoreaction product filling neuronal processes. **(A)** Fluorescent micrograph of miniSOG expressing hippocampal neurons before blue light illumination. **(B)** Phase contrast light micrograph of miniSOG-PSD-95 expressing neurons in **(A)** illuminated with blue light for 8 min at 22°C showing darkened photoreaction products filling neurons, their processes and many dots corresponding to spines and shafts (arrows, inset). Scale bar 50 μm. **(C)** Higher magnification image of a neuron and its processes after 8 min photoconversion at 22°C showing minute spines (purple arrowhead) filled with photoreaction product. Scale bar 20 μm. **(D)** Higher magnification light micrograph showing more extensive accumulation of photoreaction product after 12 min photoconversion at 22°C. **(E)** PSD-95-miniSOG expressing neurons in 40% sucrose solution at 22°C before blue light illumination. **(F)** Phase contrast light micrograph showing the same neurons as in **(E)** with photo reaction product in cell bodies and processes after 11 min photoconversion at 22°C. Arrows point to areas with numerous dark dots corresponding to PSD-95-miniSOG photoconverted spines or shafts, enlarged view in inset. **(G)** PSD-95-miniSOG expressing neurons in 60% sucrose solution at 22°C before blue light illumination. **(H)** Phase contrast light micrograph showing the same neurons in **(G)** with photoreaction product essentially in cell bodies after 12 min photoconversion at 22°C, arrow points to areas with many dark dots corresponding to photoconverted PSD-95-miniSOG in dendritic spines or shafts; enlarged view in inset.

The amount of photoreaction product generated by miniSOG is determined by the duration of photo-oxidation and the local concentration of miniSOG molecules in any particular neuronal compartment. First, we examined the time course of the photo-oxidation of miniSOG in cacodylate buffer at room temperature. The miniSOG green fluorescence appeared to photobleach rapidly, losing close to an average of ∼87% of initial intensity after ∼2 min (Figures 1H,J,K,M), and it became invisible soon after 5 min (not shown, Figure 1N at 7 min). Phase contrast images (Figures 1F,I,L,O) of the area showed emergence and accumulation of darkened material indicating DAB photoreaction product that filled cell bodies and their processes (Figures 1C,L,M,O,P), including some dot-like structures corresponding to dendritic shafts and spines where PSD-95-miniSOG was expressed in excitatory PSDs (Figure 1O). The progressive filling of dendritic processes by DAB photoreaction product was evident at 7 min (Figure 1O). Continuing illumination at room temperature for 8 and 12 min allowed further development with more pronounced accumulation and expansion of photoreaction substances filling cell bodies, fine dendritic processes and dot-like structures, some of which may correspond to PSD-95-miniSOG expressing spines and shafts (Figures 2A–D). This observation demonstrates that even with the photobleaching of visible fluorescent signals, miniSOG could continue to generate singlet oxygen under blue light illumination, thus continuing to polymerize DAB molecules, resulting in expanded filling of neural processes and structures rather than simply enhancement or enlargement of structural features already present. These results demonstrate the diffusive nature of the photoreaction products (Figures 2B–D).

**FIGURE 3:**
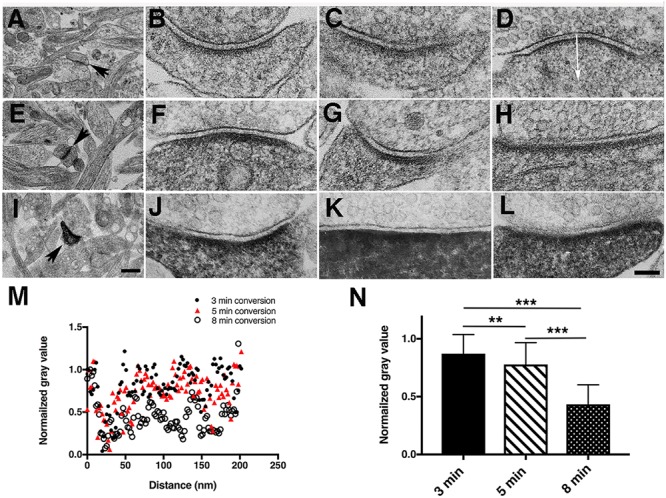
Duration of photoconversion affects the staining pattern by DAB photoreaction products in hippocampal synapses **(A,E,I)**. Low magnification electron micrographs for locating photoconverted synapses under different photoconversion times for each row, scale bar 500 nm. **(A–D)** EM micrographs of synapses after 3-min photoconversion of PSD-95-miniSOG in cacodylate buffer. **(E–H)** EM micrographs of synapses after 5-min photoconversion of PSD-95-miniSOG in cacodylate buffer. **(I–L)** EM micrographs of synapses after 8-min photoconversion of PSD-95-miniSOG in cacodylate buffer. TEM micrographs all with grid staining. All experiments at 22°C. High magnification scale bar 100 nm. **(M)** Normalized gray level line profiles from **(D,H,L)** measured vertically in the middle of a synapse starting from the presynaptic membrane as indicated by the arrow in **(D)** across the synaptic cleft into the postsynaptic terminal showing prolonged photoconversion time correlated with progressive darkening of cytoplasm in dendrites or shafts due to DAB photoreaction product staining. **(N)** Bar graph comparing the mean of the normalized gray level measured 40 to 200 nm from the presynaptic membrane into the cytoplasm of dendrites or shafts of synapses at three different photoconversion times in **(M)** showing that the gray levels are quantitatively differentiable: 0.87 ± 0.16 (3 min, mean ± SD, *n* = 83), 0.78 ± 0.19 (5 min, *n* = 83), 0.43 ± 0.17 (8 min, *n* = 81), *P* = 0.002,^∗∗^(3 min vs. 5 min); *P* < 0.0001,^∗∗∗^(3 min vs. 8 min); *P* < 0.0001, ^∗∗∗^(5 min vs. 8 min), one way-ANOVA, Tukey’s multi-tests.

**FIGURE 4:**
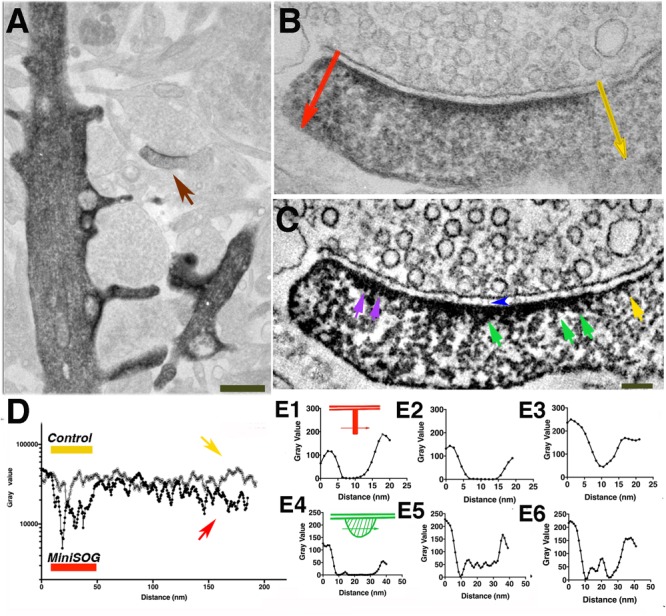
Electron microscopy of PSD-95-miniSOG expressing neurons photoconverted for 8 min in cacodylate buffer at 22°C. **(A)** Survey electron micrograph shows examples where PSD-95-miniSOG expressing in neuronal processes and synapses are more electron-dense and readily distinguishable from those in non-transfected surrounding neurons following photoconversion with grid stain. Brown arrow points to a dendritic spine expressing PSD-95-miniSOG. Scale bar, 1 μm. **(B)** Higher magnification TEM image of the synapse indicated by the arrow in **(A)** showing PSD-95-miniSOG expressed in its dendritic spine. Red vector line indicates the path along which an intensity line profile in DAB stained region is plotted as solid dark dots in **(D)** Yellow vector line shows where an intensity line profile was plotted in a control region lacking DAB staining, plotted as open circles in **(D)**. **(C)** Virtual section (1.4 nm thick) of the synapse in **(B)** showing that the aggregates of DAB photoreaction product staining various structures (e.g., membrane-associated vertical filament indicated by purple arrows, membrane-associated bulky structure indicated by green arrows) at the PSD extend throughout the cytoplasm of the dendritic spine. A membrane-associated vertical filament not stained by the photoreaction product as a control structure indicated by yellow arrow. Transcleft filament indicated by a blue arrowhead. Scale bar 100 nm. **(D)** Quantitative comparison of intensity line profiles in a DAB stained region (black dots, red arrow) vs. that from a control region without DAB staining (open dots, yellow arrow) in **(B)**. Structural details revealed by the line profiles include thickness of postsynaptic membrane: 8 nm for DAB region (FWHM of the first sharp trough) and 6 nm for control region (FWHM of the first sharp trough). PSD thickness: 33 nm from FWHM of along a wide though in the DAB stained region. The averaged intensity along the path lines at distances larger than 80 nm from the postsynaptic membrane was 23219 ± 4829 (DAB stained region, *N* = 182) vs. 36535 ± 5360 for the control region. (*N* = 182, *p*<0.0001, Student’s *t*-test). **(E1–E5)** Size of structural elements at the PSD determined from tomogram of the photoconverted synapse in **(B)**. Diameter of vertical filament in the DAB stained region indicated by purple arrows in **(C)** from left to right determined by FWHM of line profiles: 10 nm **(E1)**, 13 nm **(E2)**. Diameter of vertical filament in the control region indicated by the yellow arrow in **(C)** as determined by the FWHM of line profile: 7 nm **(E3)**. Size of the membrane-associated bulky structures measured from line profiles running parallel to and ∼10 nm away from the postsynaptic membrane was 28 nm **(E4)**, 29 nm **(E5)**, and 23 nm **(E6)**.

Some dark dots may correspond to dendritic processes or shafts where PSD-95-miniSOG was expressed in the PSDs of excitatory synapses (Figure 2B and inset, Figures 2F,H and insets) at the light microscopy level following photoconversion. We used electron microscopy to identify these structures. We employed a diamond pointer mounted on the inverted microscope objective stage to etch a ∼1.6 mm diameter circle marking the area containing neurons and processes to be photoconverted (Figures 1D,P), so the etched mark on the glass encircled the photobleached neurons (Supplementary Figure S1). After photoconversion, the coverslip in the DAB solution was immediately put on ice, treated with osmium solution (0.25–1%) and then dehydrated, followed with a modified EM embedding protocol in which supplemental heavy metal staining was omitted (for details see “Materials and Methods”) to avoid obscuring electron-dense signals from the osmicated DAB reaction product. The staining created by miniSOG precipitates alone, however, offers little contextual contrast under conventional transmission EM (TEM) (Figures 6A,D,G, 8A), so it is typically necessary to use grid staining for conventional EM analysis of TEM images of photo converted synapses (Figures 3A–L, 4A,B).

### Electron Microscopy of MiniSOG Positive Synapses

Photoconverted neurons were localized in the plastic embedded block by following the etched circle on the glass coverslip, and the area was then cut out and mounted on an ultramicrotome for thin sectioning. For the initial examination of the consequences of miniSOG photoreactions by TEM, each grid was conventionally stained with uranyl acetate and lead citrate. Morphologies of synapses in the 3-min photoconversion group at room temperature (11 synapses) were not significantly different from nontransfected synapses except there was a hint of slightly more densely stained cytoplasm and PSDs in spines (Figures 3A–D). At 5 and 8 min of photo-oxidation, the DAB photoreaction produced fine electron-dense staining in electron micrographs that filled the entire cytoplasm of dendritic spines, where it heavily decorated proteins, especially the postsynaptic density (PSD). The 5-min group (29 synapses, 9 dual-axis tomograms) showed prominent electron-dense staining of PSDs and cytoplasmic structures in dendritic spines and shafts (Figures 3E–H). Synapses in the 8-min photoconversion group (77 synapses, 3 dual axis tomograms) often had far more extensive electron-dense staining of PSDs and cytoplasmic structures, to the point that some of the detailed cytoplasmic fine structures were obscured in thin section TEM micrographs (Figures 3I–L). Quantitative analysis of normalized line profiles under these conditions confirmed these observations (Figures 3M,N).

**FIGURE 5:**
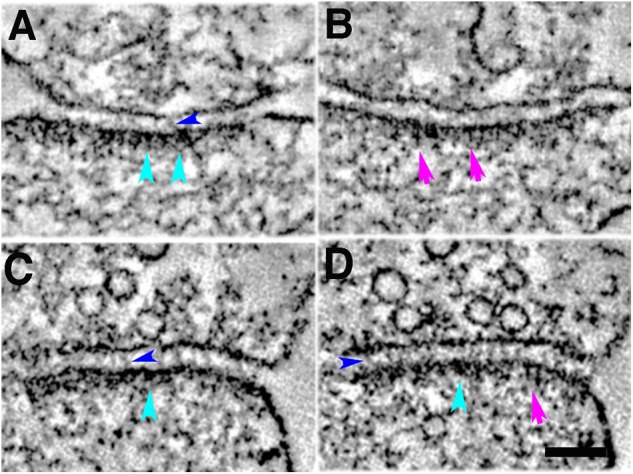
Five-minute photoconversion reaction of MiniSOG in cacodylate buffer at 22°C delineates two main types of structural elements at the postsynaptic density in tomograms: membrane-associated cytoplasmic bulky structures at the PSD (cyan arrowheads, **A,C,D**); membrane-associated vertical filaments at the PSD (purple arrows, **B,D**); trans-cleft filaments (blue arrowheads, **A,C,D**). Sample was grid stained. Scale bar 100 nm.

Analysis of gray values of electron micrographs from line profiles of TEM images collected at 8-min photoconversion at room temperature allowed quantitative differentiation between DAB stained regions and control regions without DAB staining in spine cytoplasm. (Figure 4D). Line profiles also revealed distinct fine structural features such as the postsynaptic membrane, PSD and cytoplasmic elements (Figure 4D). EM tomography of the same synapse (Figure 4B) revealed several classes of structures in the PSD, such as membrane-associated vertical filaments (Figure 4C), a main structural feature of the PSD first seen by freeze fracture, deep etching electron microscopy in auditory synapses (Gulley and Reese, 1981) and then seen in tomograms of intact synapses in freeze-substituted hippocampal cultures (Chen et al., 2008b). However, with 8-min photoconversion at room temperature, the diameters of these vertical filaments, as determined from full widths at half maximum (FWHM) of the line profiles were 10–13 nm (Figures 4E1,E2), i.e., significantly larger than the 7 nm diameter of the control vertical filament in the same tomogram without DAB staining (Figure 4E3). This result was consistent with the diameter of 5–6 nm for the typical vertical filaments, as previously reported (Chen et al., 2008b), which suggested decoration by electron-dense photoreaction products during prolonged photo-oxidation. Also present is a second major class of PSD-associated structures, which we termed “membrane-associated bulky structures,” each often corresponding to transcleft filaments previously reported to comprise several types of cleft adhesion molecules bridging the synaptic cleft (High et al., 2015) (Figure 4C). These structures apparently represent decorated versions of the cytoplasmic side of the transmembrane structures seen in intact PSDs in freeze-substituted hippocampal synapses. Some of these structures may correspond to the cytoplasmic side of glutamate receptors (Chen et al., 2008b). In addition, some larger transmembrane structures were seen with “waist length” FWHM values of 23–28 nm, implying that their length along the membrane is 46–56 nm (Figures 4E4–E6), i.e., significantly wider than the typical ∼20 nm extensions along the membrane of the membrane-associated bulky structures (Chen et al., 2008b, 2011, 2015), assuming an accumulation of photoreaction product that enlarged them (Figure 4C). For comparison, we classified corresponding core-membrane structural elements in PSDs from 5-min photoconversion tomograms, measuring diameters of vertical filaments and the size of membrane-associated bulky structures (Figure 5). The diameters of vertical filaments determined from a line profile of FWHM was 5.1 ± 0.4 nm (*N* = 5) (Figures 5B,D), and the length along the membrane of the bulky structures was∼20 nm (Figures 5A,C,D), much lower than those in the 8-min group (Figures 4C,E) but very close to the sizes of these type of structures in PSDs from tomograms of freeze-substituted synapses (Chen et al., 2008b, 2011, 2015). These differences reinforce the interpretation that DAB precipitates have decorated and thickened these structural elements in PSDs. Overall, the two main structural elements in the PSDs seen in these tomograms are consistent with what we have found by analyzing freeze-substituted synapses, and the increase in size of vertical filaments and bulky membrane-associated structures suggest decoration by electron-dense photoreaction products (Figures 4C, 5).

### Reducing Molecular Diffusion Restricts Spreading of DAB Photoreduction Product During Photo-Oxidation

Suspecting that the DAB photoreaction product is very diffusive, we reasoned that the ubiquitous presence of miniSOG photoreaction staining in synapses is likely due to diffusion of DAB photoreaction products away from miniSOG molecules fused to PSD-95. In that case, it might be possible to localize PSD-95 by visualizing signals from PSD-95-miniSOG photoreaction products, if we were able to increase the local viscosity to limit the molecular diffusion. We tested this possibility by incorporating sucrose into the reaction buffer solution at 22°C, using different weight % concentrations of sucrose to change the viscosity η (given in units of centipoise, cP): 20% (w/w, viscosity of 1.9 cP, 22°C), 30% (3 cP), 40% (5.7 cP), and 60% (52 cP) sucrose solutions (Swindells et al., 1958). We also tested lowering the temperature of the sucrose-containing incubation medium during the entire photoconversion process using a temperature-control device fitted onto the water immersion objective lens and cooled by circulating water from a temperature controlled water bath set to 0.4°C leaving the local temperature at the specimen ∼4°C. Lowering the temperature increases the viscosity of the medium by factor of ∼2 (Swindells et al., 1958). According to the Einstein relationship $D=\frac{kT}{6\pi \eta a}$, where D is the diffusion coefficient, *a* is radius of DAB molecule, *η* is the local viscosity of the medium, *k*, the Boltzmann constant, *T* temperature (Berg, 1983), the change in temperature from 295 K (22°C) to 277 K (4°C) only represents a 6% change, but the diffusion coefficient decreased significantly due to increase of viscosity. Therefore, lowering temperature could markedly reduce the diffusion of DAB photoreaction product away from the proximity of miniSOG, thereby generating more localized signals reflecting the location of miniSOG molecule on PSD-95.

At the light microscopy level, the presence of sucrose in the photoconversion medium at room temperature restricted the photoreaction material largely to the cell body (Figures 2E–H): only weak signs of photoreaction product were evident in fine dendritic processes in the 40% sucrose (Figure 2F) and very little signal was seen in neuronal processes in the 60% sucrose solution (Figure 2H), though dark dots presumably corresponding to dendritic spines or shafts filled with photoreaction product are evident in all preparations, likely due to photoconverted PSD-95-miniSOG in PSDs of excitatory synapses (insets Figures 2B,2F,H). A significant amount of photoreaction product can be seen after just 2 min of photo-oxidation (Figure 1L) in cacodylate buffer at room temperature (viscosity, 1 cP). In a sharp contrast, the photoreaction time in sucrose solution at room temperature needs to be significantly lengthened to ∼10 min (8–12 min) to see similar levels of darkened reaction products (Figures 2F,H). We interpret this difference to reflect that the presence of sucrose solution limits the spreading of DAB reaction product. Additional factors include the possibility that viscous sucrose solution could reduce the number of DAB molecules entering into the vicinity of miniSOG molecule, or even that presence of sucrose might affect the photophysics of miniSOG, could also contribute to this effect.

**FIGURE 6:**
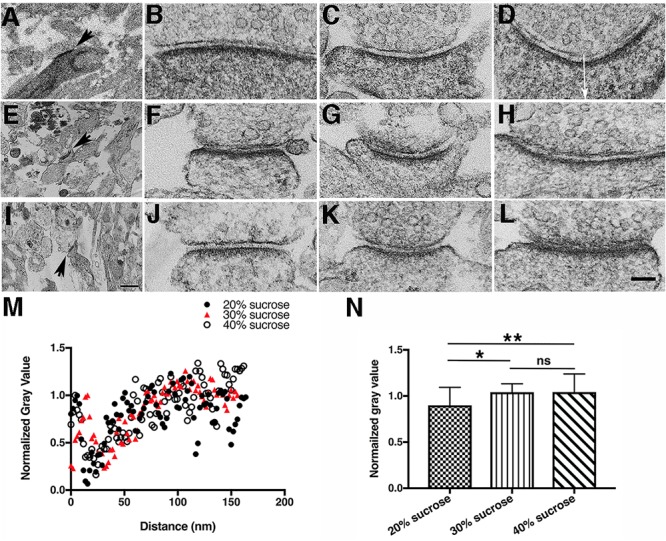
Increasing sucrose concentration affects DAB photoreaction product staining pattern in hippocampal synapses. **(A,E,I)** Low magnification electron micrographs for locating photoconverted synapses under different sucrose concentrations for each row, scale bar 500 nm. **(A–D)** Photoconversion of PSD-95-miniSOG in 20% (w/w) sucrose solution, 9-min photoconversion time. **(E–H)** photoconversion of PSD-95-miniSOG in 30% (w/w) sucrose, 9-min photoconversion time. **(I–L)** Photoconversion of PSD-95-miniSOG in 40% (w/w) sucrose, 11 min photoconversion time. **(M)** Normalized line profiles from **(D,H,L)** measured vertically in the middle of a synapse starting from the presynaptic membrane as shown by the arrow in **(D)** into the cytoplasm of the postsynaptic terminal showing increased sucrose concentration reduces spreading of DAB photoreaction product staining in the cytoplasm. All experiments at 22°C. High magnification scale bar 100 nm. **(N)** Bar graph comparing the mean of the normalized gray level measured 52 to 164 nm from the presynaptic membrane into the cytoplasm of the dendrites or shafts of synapses in 20, 30, and 40% sucrose solutions in **(M)**. The normalized gray levels due to DAB staining are quantitatively differentiable in higher concentration sucrose solutions (30 and 40%) vs. in lower concentration sucrose solution (20%): 0.88 ± 0.18 (20% sucrose, *n* = 59), 0.98 ± 0.16 (30% sucrose, *n* = 55), 1.00 ± 0.22 (40% sucrose, *n* = 58), *P* = 0.02,^∗^(20% vs. 30%); *P* = 0.0046,^∗∗^(20% vs. 40%); *P* = 0.88, ns, (30% vs. 40%). All normalized gray scale was calculated by dividing original gray values in line profile with a maximum gray value within 25 nm from the presynaptic membrane.

Under electron microscopic examination, the DAB staining varied somewhat from one synapse to another, but a general trend was evident: in synapses in 20% sucrose solution (30 synapses) at room temperature, the DAB staining is still pervasive at PSDs and in cytoplasm of the spines or shafts (Figures 6A–D). However, in 30% (45 synapses) (Figures 6E–H) and 40% (44 synapses) (Figures 6I–L) sucrose solutions, the DAB staining is largely restricted to PSDs, not significantly spreading to cytoplasm of spines or shafts as documented by the quantitative analysis of normalized line profiles in these sucrose solutions (Figures 6M,N).

### Identification of PSD-95 Filaments at the PSD Based on Location of DAB Clusters Generated by PSD-95-MiniSOG

**FIGURE 7:**
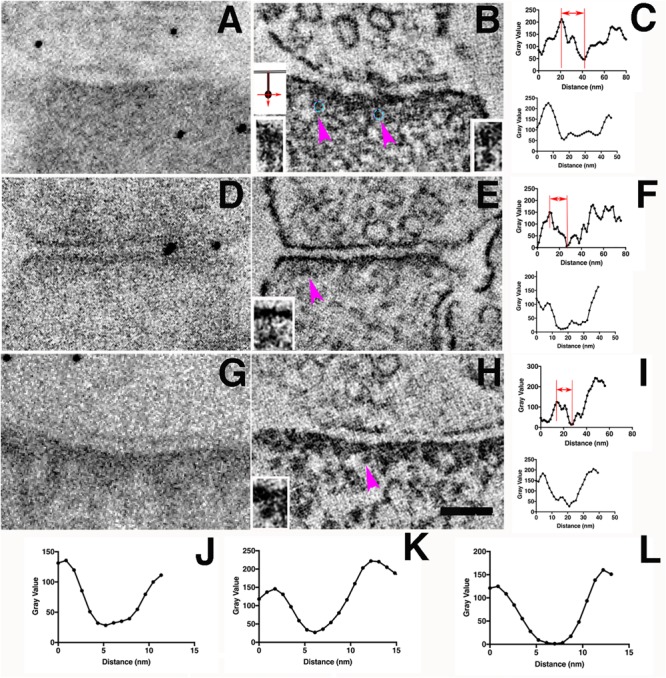
Electron-dense clusters indicative of miniSOG are revealed by EM tomography. **(A)** Transmission electron micrograph (no grid staining, 300 kV) of a synapse positively expressing PSD-95-miniSOG photoconverted in 60% sucrose at 22°C for 12 min. **(B)** Virtual section of the synapse in **(A)** showing examples of prominent electron-dense clusters (cyan circles) at the distal ends of membrane-associated vertical filament indicated by purple arrowheads **(B,E,H)**. Upper left inset showing schemes of two orthogonal ways of measuring the line profiles across the electron- dense cluster as indicated by purple arrows in **(B,E,H)**. The vertical line profile starts at the postsynaptic membrane then passes the electron-dense structure deeper into the cytoplasm of the synapse while the transverse line profile centered around the electron-dense structure perpendicular to the vertical line. Insets at the bottom corners in **(B,E,H)** showing magnified view from tomograms of the membrane-associated vertical filament with its distal end electron-dense cluster **(C,F,I)**. Top, Vertical line profile showing the location of electron-dense cluster; Bottom, Transverse line profile across the electron-dense cluster in **(B,E,H)**. The estimated distance of approximate center of the electron-dense cluster to the postsynaptic membrane: 20 nm (**B**, left), 22 nm (**B**, right, line profile not shown),16 nm **(E)**,14 nm **(H)**. FWHM of transverse line profiles across electron-dense clusters for estimating their diameters: 30 nm (**B**, left), 21 nm **(E)**, 23 nm **(H)**. **(D)** Transmission electron micrograph (no grid staining, 300 kV) of a synapse positively expressing PSD-95-miniSOG photoconverted in 40% sucrose at 22°C for 11 min. **(E)** Virtual section of the synapse in **(D)** showing examples of electron-dense structure at the distal end of membrane-associated vertical filament (purple arrowhead, inset). **(G)** TEM micrograph (no grid staining, 300 kV) of a synapse positively expressing PSD-95-miniSOG photoconverted in 40% sucrose at 4°C for 9 min. **(H)** Virtual section of the synapse in **(G)** showing examples of prominent electron-dense cluster at the distal end of membrane-associated vertical filament (purple arrowhead, inset). Scale bar 100 nm. All virtual section are averaged in 3–5 neighboring frames.**(J–L)**. Transverse line profiles across vertical filaments for estimating the diameter of vertical filaments associating with the electron-dense cluster: 5.8 nm (**B**, left), 5.4 nm **(E)**, 6.7 nm **(H)**. **(A,D,G)** Black dots are 10 nm gold particles used as fiducial markers for calculating tomograms.

**FIGURE 8:**
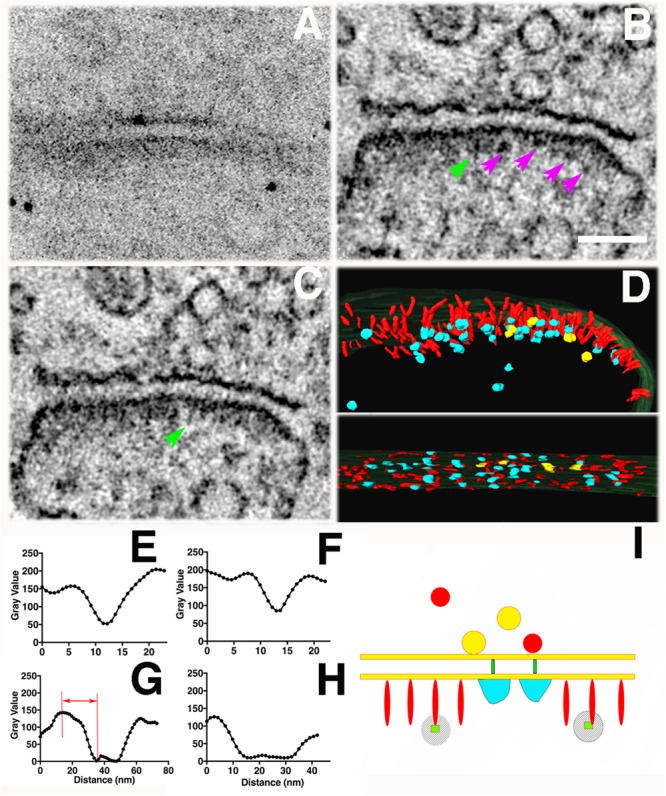
Tomography of a synapse where photoconversion of PSD-95-miniSOG was performed at room temperature (22°C) and in the presence of 40 % sucrose for 11 min. **(A)** TEM micrograph of a PSD-95-miniSOG positive synapse without grid staining (300 kev), black dots are ∼10 nm gold particles used as fiducial markers for calculating tomogram. **(B)** The DAB photo reaction product appears as a series of electron-dense clusters (purple arrows) arranged transversely at an approximately similar distance from the PSD membrane in tomogram of **(A)**, vertical filament at the PSD indicated by a green arrow. Scale Bar 200 nm. **(C)** Virtual section of the synapse in **(A)** showing an array of densely decorated filaments (green arrow) lining the postsynaptic membrane. **(D)** Surface rendering of virtual sections from tomogram of **(A)** from two views showing the membrane-associated filaments and their electron-dense clusters at the PSD. Cross section view (top) shows that the ends of vertical filaments (red) are decorated by aggregates of dense material (cyan), except that those indicated by purple arrows in **(B)** are colored yellow. Some electron-dense cluster-like structures are distributed further away from the PSD core deeper in the cytoplasm of the spine. In *en face* view (bottom), the aggregates of dense material appear to be regularly arrayed along the postsynaptic membrane at the PSD. The labeling pattern of miniSOG in association with vertical filaments in the PSD matches the known distribution of PSD-95 in the PSD, further confirming that the membrane-associated vertical filaments contain PSD-95 at the PSD. **(E)** Line profile perpendicular to the vertical filament indicated by green arrow in B, FWHM estimation of the diameter of vertical filament to be 5 nm. **(F)** Transverse line profile perpendicular to the vertical filament indicated by green arrow in **(C)**, FWHM estimation the diameter of vertical filament to be 5.8 nm. **(G)** Vertical line profile for estimating the distance of the electron-dense cluster in **(B)** to the postsynaptic membrane: 25 nm (first left). **(H)** A transverse line profile for estimating the diameter of electron-dense cluster in **(B)**: 20 nm (first cluster from the left indicated by purple arrow). Sizes of other clusters measure from line profiles (not shown) from left to right all indicated by purple arrows in **(B)**: 20 nm, 15 nm, 20 nm. **(I)** Schematic of detailed structural elements in a PSD-95-miniSOG overexpressing synapse revealed by EM tomography. PSD-95-like vertical filament (red), miniSOG (green square) are surrounded by electron-dense DAB precipitates (stripes shadow), bulky membrane- associate structures (cyan), trans-cleft filaments (green). Red and yellow circles correspond to presynaptic vesicles.

Having successfully limited diffusion of DAB staining by incubation in sucrose solutions, we explored whether molecular localization of miniSOG might be feasible by electron microscopy. We predicted that the presence of a highly viscous reaction medium might allow more polymerized DAB molecules to accumulate locally around a miniSOG molecule, where it could act as a nucleating center for accumulating even more DAB polymer. To test this, we needed to image localized DAB photoreaction products at high resolution. Conventional TEM micrographs represent a superposition of overlapping small structures within a typical section thickness of ∼80 nm. Individual molecules or complexes only a few nanometers in size are engulfed in a thicket of other proteins, precluding definitive analysis. Accordingly, dual-axis TEM tomography, which definitively overcomes the overlap problem, was used to make 3D reconstructions of PSDs that express miniSOG. These synapses were devoid of grid staining so that the electron-dense material represents only the distribution of the photoreaction products, except for membrane lipids and other strongly osmiophilic endogenous components of the cell. We examined tomographic reconstructions of miniSOG expressing synapses photoconverted 8–12 min in 40 or 60% sucrose solutions at room temperature (22°C) (Figures 7A–F, 8A–D) and at low temperature (4°C) in 40% sucrose solution (Figures 7G–I). We searched these tomograms for electron-dense structures at the distal ends of vertical filaments in PSDs. The diameters of the vertical filaments are 5–7 nm (Figures 7J–L) as expected.

Among eleven tomograms from three different photoconversion experiments at both room temperature and low temperature, at least four tomograms contained individual examples where a prominent electron-dense cluster could be located at the distal end of a membrane-associated vertical filament at the PSD (Figures 7B,E,H and insets) and in one example a row of vertical filament-associated clusters was found in the tomogram (Figures 8B,D). These electron-dense clusters, with diameters of 15–20 nm (Figure 8H), are located at an average distance of 21 ± 3 nm (*N* = 17, measured from the surface rendering model in Figure 8D) or 25 nm (Figure 8G) from the postsynaptic membrane, fitting very closely with the predicted location of the miniSOG molecule itself at the extreme *C*-terminal end of the extended PSD-95 molecule, which we estimate as ∼20 nm. At the PSD, PSD-95 has been identified with EM tomography and by immunolabeling of its *N* and *C* terminal ends (Chen et al., 2008b). It appears that PSD-95 *N*-termini are at the membrane and *C*-termini are away from the membrane (Chen et al., 2011, 2015; Jeyifous et al., 2016). Molecular manipulation of the AMPAR auxiliary unit of the Stargazin tail which binds to PSD-95, combined with imaging analysis further confirmed the vertical configuration of PSD-95 at the PSD (Hafner et al., 2015). Thus, the *C*-terminally fused miniSOG would be expected to be located ∼20 nm away from the postsynaptic membrane at the PSD. Considering that a typical extended PSD-95 filament is ∼17-nm long and miniSOG, which is half the molecular mass of GFP, should add an additional ∼3 nm to the length. Thus, the center of miniSOG is predicted to be ∼17 + 2 = 19 nm from the postsynaptic membrane. Surface rendering of the tomogram showed that a cluster is located at the distal end of individual membrane-associated vertical filaments (diameter 5–6 nm, Figures 8E,F), demonstrating that some of the membrane-associated vertical filaments contain PSD-95 (Figure 8I). We compared the locations of the dense clusters coding for miniSOG with that of the PSD-95-EYFP site determined by immunogold labeling (first with antibody against GFP, then with a Fab-conjugated secondary attached to Nanogold (1.4 nm) and subsequently silver-enhanced). This preparation gave an average distance from the center of the gold particle to the membrane of 27 ± 7 nm (Chen et al., 2011) (*N* = 326, *p* < 0.0001, Student *t* test, compared with miniSOG electron cluster distance to the membrane). Therefore, the location of miniSOG site to the postsynaptic membrane is ∼6 nm closer than the immunogold site, in line with the expectation that the presence of primary and secondary antibody complexes in the latter scenario added extra separation from the target sites. With the capacity to visualize an electron-dense cluster and a molecular structure in its close proximity, our approach allowed us to identify miniSOG tagged PSD-95 at the PSD. Thus we conclude that miniSOG has the potential to be a GFP-like reporter for electron microscopy to localize expressed moieties of proteins in synapses.

## Discussion

MiniSOG is a potent singlet oxygen generator, which–when activated by blue light (Shu et al., 2011) – results in oxidation of diaminobenzidine (DAB) in the incubation medium. The resulting DAB polymers bind OsO_4_ at its amino groups, thus generating an amorphous electron-dense material in electron micrographs. Unfortunately, some of the activated DAB diffuses away before it polymerizes (Courtoy et al., 1983), so not all of the photoreaction product seen in electron micrographs represents, at the molecular level, the exact locations of the expressed singlet oxygen generators. To complicate the picture further, the unquenched singlet oxygen, with an average life time of 3–4 μs in cells, may diffuse about two hundred nanometers away from its source (Skovsen et al., 2005), creating additional background photoreaction intermediates.

The very first *C*-terminal PSD-95-GFP construct, when overexpressed in neurons, was specifically localized at the postsynaptic side of excitatory synapses in cultured hippocampal (Craven et al., 1999) and in cortical neurons (Arnold and Clapham, 1999). Overexpression of PSD-95-GFP recruits more AMPARs to synapses, resulting in enlargement and maturation of excitatory synapses (El-Husseini et al., 2000), and promoting synaptogenesis in cultured hippocampal neurons (Nikonenko et al., 2008). The specificity of PSD-95-GFP expression in excitatory dendritic spines and shafts was also replicated in hippocampal brain slices (Schnell et al., 2002). The distribution of PSD-95-GFP in the PSD has been extensively studied by immunolabeling, TEM tomography and molecular manipulations (Chen et al., 2008b, 2011, 2015; Hafner et al., 2015; Levy et al., 2015; Jeyifous et al., 2016). Here, we first made a miniSOG tagged PSD-95 construct by swapping *EYFP* at the C-terminal end of PSD-95-EYFP with *miniSOG*. The miniSOG (13.9 kD) is much smaller than EYFP (26.4 kD), which might improve specificity of labeling. The first question addressed in the present work was how the distribution of a miniSOG moiety fused PSD-95 compared with the known distribution of PSD-95 at the PSD determined by other methods (DeGiorgis et al., 2002; Petersen et al., 2003; Chen et al., 2008b, 2011, 2015; Farley et al., 2015). In our hands, the specificity of PSD-95-miniSOG to postsynaptic side of synapses was evident in light micrographs in the numerous black dots corresponding to spines or shafts of excitatory synapses in all photoconversion experiments. In electron micrographs, essentially all PSD-95-miniSOG-expressing synapses showed prominent electron-dense PSDs consistent with the expectation that overexpressed PSD-95-miniSOG molecules are present in these PSDs.

At room temperature, we documented that the duration of photoconversion time, which determines the number of incoming blue photons and, likely, determines how many singlet oxygen molecules are generated to oxidize DAB molecules, was the principal factor in generating DAB signal for EM. These results indicate that miniSOG provides an excellent synaptic tag to label individual miniSOG-expressing neurons, since electron-dense staining from photoconversion products fill transfected dendrites and their spines. The diffusive spread of the DAB photoreaction product, however, prevents specific localization and identification of individual molecules in synapses.

Localization by photoconversion of DAB is limited by diffusion of the DAB photoreaction product (Courtoy et al., 1983); early work showed that the amount of diffusion can be decreased, but only to a limited degree (Courtoy et al., 1983; Brown and Farquhar, 1989), and prolonged photoconversion created more diffusive photoreaction product that spread throughout neuronal processes. We therefore explored ways to reduce the diffusion of oxidized DAB away from miniSOG moieties by using sucrose solutions, which increase the viscosity of the medium and may therefore hinder spreading of DAB photoreaction product, should it cross the membrane into the cytoplasm of cells. At high sucrose concentrations (which exhibit high viscosity), the DAB signal was essentially limited to the cell body and to dot-like structures corresponding to photoconverted spines or shafts of excitatory synapses. Although dampening diffusion of DAB molecules could account for the significant lengthening of photoconversion times to achieve comparable visible results, viscous media may also reduce the number of DAB molecules available for photoconversion and for quenching the diffusive singlet oxygen (Kuimova et al., 2009) inside the neuron. At the TEM level, 8 min of room temperature photoconversion in cacodylate buffer produced thick DAB staining filling the entire cytoplasm of spines, whereas 10 min conversion in 20% sucrose solution resulted in heavily stained PSDs and considerably less staining of the cytoplasm. With 30 and 40% sucrose at room temperature, the DAB staining was largely limited to the PSD with very little spreading to other cytoplasmic structures. We concluded that increasing viscosity with sucrose restricts the DAB staining to the PSD, whereas photoconversion in buffer without sucrose solution the reaction product may spread up to several hundred nanometers into the cytoplasm.

Sucrose cannot cross intact cell membranes by diffusion alone, but aldehyde fixed cells are freely permeable to sucrose (Griffiths et al., 1984), and this effect was critical for vitrifying tissues in the Tokuyasu method for immunogold labeling of cryosections (Tokuyasu, 1973; Bos et al., 2014). In our photoconversion experiments involving sucrose solutions, DAB was dissolved in sucrose solutions first and exchanged for a period of time before onset of photoconversion. The resultant photoconversion showed that DAB went across membrane into neuronal cell bodies and processes with concomitant effects of dampening diffusion of DAB photoreaction product in higher concentration of sucrose solutions. Furthermore, cells fixed with glutaraldehyde showed less change in cell sizes due to osmotic stresses than do cells fixed with OsO_4_ (Penttila et al., 1974). Considering also that we have not noticed significant morphological changes in cells or in synapses, our results are better accounted for by local viscosity changes than by osmotic effects.

The successful demonstration of the effectiveness of the viscous medium in limiting diffusion in photoconversion led us to explore means to localize miniSOG more precisely. We used 40% or 60% sucrose at room temperature, as well as lowering the temperature from 22 to 4°C to further reduce diffusion during the photoconversion in 40% sucrose solution. To detect the photoreaction signal reliably, we imaged synapses without grid staining, using the detailed 3D structural information provided by EM tomography to determine the exact distribution of the DAB photoreaction product at the PSD. This allowed detection of electron-dense structures at the distal ends of the membrane-associated vertical filaments at PSDs, identified as PSD-95-miniSOG.

Either increasing the viscosity of medium, cooling the incubation medium, or combination of both, can effectively reduce diffusion (Purcell, 1977; Berg, 1983). For example, one could use 60% sucrose solution at room temperature, which is very viscous and difficult to handle, or combining the two approaches using much less viscous 40% (w/w) sucrose but at low temperature to further increase viscosity of the medium. Lowering the temperature by close to 20°C would double the viscosity of the sucrose solution (Swindells et al., 1958), thus thermal diffusion of oxidized DAB should be significantly curtailed in sucrose media and at low temperature.

In addition, our temperature reduction (∼6% reduction in terms of *kT*) likely had little effects on miniSOG photophysics in terms of the rates of singlet oxygen releases. Indeed, from 23 to 10°C, quantum efficiency of miniSOG and lifetime of singlet oxygen from miniSOG only increase slightly, 7 and 6%, respectively (Westberg et al., 2017).

With the high viscosities that we could muster with sucrose at room and lower temperature, the diffusion of photoreaction products was markedly constrained. Indeed, this improvement appears to provide reliable indication of the location of the PSD-95 molecules as marked by miniSOG constructs. Upon closer examination by tomography, it was evident that some diffusion still occurred on the scale of individual proteins. However, concentrations of DAB photoreaction product appearing as electron-dense clusters at the tips of the membrane-associated vertical filaments in the PSD, known to contain PSD-95, indicate that it is feasible to localize miniSOG-labeled protein/complex in place without the complications introduced by immunogold antibody complexes, provided their individual molecular structure is well defined. The antibody complex may appear as a filamentous structure, potentially confounding the structural interpretation of tomograms (Chen et al., 2008a,b, 2011).

Through this work, we uncovered other properties of miniSOG. For example, we observed that continuous illumination with blue light continuously generates more DAB photoreaction products, which fill more neuronal processes, suggesting that miniSOGs is continuously generating singlet oxygen beyond photobleaching. This finding is consistent with a recent study showing that accumulative illumination of miniSOG could significantly increase its quantum yield (up to 10-fold) likely due to miniSOG protein transformation during light illumination (Ruiz-Gonzalez et al., 2013).

The precision of the miniSOG localization might be further improved to the level of individual proteins by further increasing the viscosity of medium in combination with a lower temperature for the DAB photoreaction. However, several caveats might affect its general application. First, cross-linking chemical fixation is required to stabilize tissue during incubation, though results are often surprisingly close to those in directly frozen freeze-substituted tissues. Second, additional heavy metal staining on sections must be avoided in order not to have other stained objects competing with the osmicated DAB. Most importantly, the protein chosen to be tagged with miniSOG should have relatively well defined molecular structure, for example individual PSD-95 and SAP97 molecules have been imaged by single particle EM (Nakagawa et al., 2004), allowing these structures to be used as references to compare with structures labeled by miniSOG electron-dense clusters in tomograms of synapses. In conclusion, we have demonstrated that overexpression of miniSOG tagged protein in neurons, and then photoconverting DAB in viscous sucrose medium at room or lowered temperature can be useful to identity prevalent molecules in synapses when imaged by EM tomography (Nakagawa et al., 2004; Chen et al., 2008b, 2015). The methods may also improve localization of proteins tagged with other peroxidase-based constructs using DAB reactions, such as APEXs, (Martell et al., 2012; Lam et al., 2015).

## Author Contributions

XC and TR designed the research and wrote the paper. XC, CW, VC, and AS performed the research. RL contributed to the electron microscopy. ML contributed a new reagent.

## Conflict of Interest Statement

The authors declare that the research was conducted in the absence of any commercial or financial relationships that could be construed as a potential conflict of interest.

## Supplementary Material

FIGURE S1 Low magnification fluorescence image of PSD-95-miniSOG expressing neurons after photoconversion. The dark circle in the middle corresponds to an area which was photobleached. Arrow pointing to the scratch mark by the diamond edge which often encircle the photobleached area. Scale bar 1 mm.
