# Supplementary figures and images for: Identification of PSD-95 in the Postsynaptic Density Using MiniSOG and EM Tomography
Source: Front Neuroanat. 2018 Dec 7;12:107. doi: 10.3389/fnana.2018.00107 (PMC6292990; doi:10.3389/fnana.2018.00107)

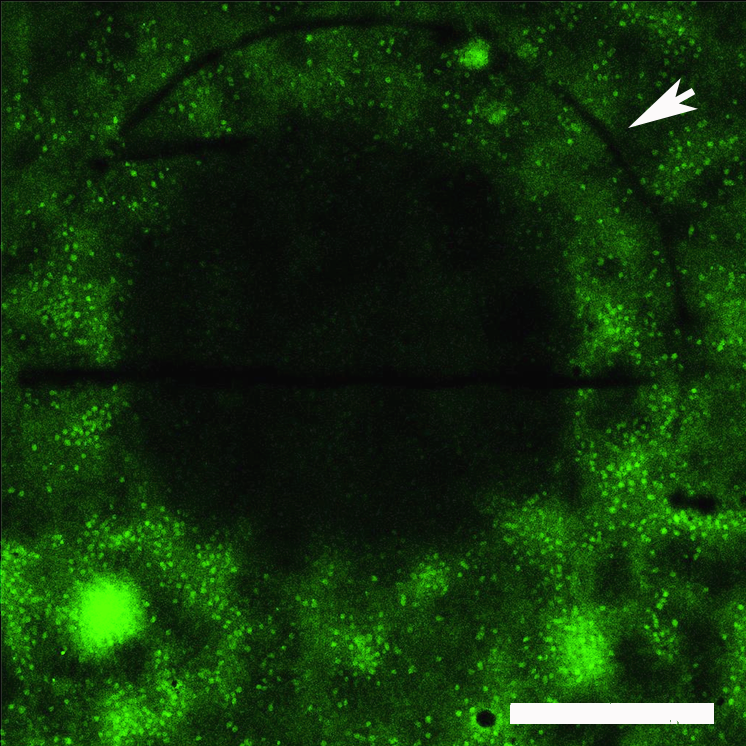

Supplement: FIGURE S1 — Low magnification fluorescence image of PSD-95-miniSOG expressing neurons after photoconversion. The dark circle in the middle corresponds to an area which was photobleached. Arrow pointing to the scratch mark by the diamond edge which often encircle the photobleached area. Scale bar 1 mm. [file Image_1.TIF]
